# Multiple Congenital Granular Cell Epulis: Case Report and Immunohistochemical Profile with Emphasis on Vascularization

**DOI:** 10.1155/2015/878192

**Published:** 2015-02-05

**Authors:** Patricia Roccon Bianchi, Vera Cavalcanti de Araujo, José Wagner Banterli Ribeiro, Fabricio Passador-Santos, Ney Soares de Araujo, Andresa Borges Soares

**Affiliations:** Department of Oral Pathology, São Leopoldo Mandic Institute and Research Center, 13045-755 Campinas, SP, Brazil

## Abstract

Congenital granular cell epulis is a rare benign soft tissue lesion arising from the alveolar ridge in neonates. A rare case of multiple congenital granular cell epulis is reported, alongside a description of its vascular immunohistochemical profile. A female newborn presented with two exophytic pedunculated red nodules located on the alveolar ridge between the future eruption sites of the incisors and canines of the mandible and maxilla. A conservative surgical excision was performed on the second day of life. Histology revealed proliferation of round granular cells containing an abundant eosinophilic cytoplasm with basophilic nuclei, ranging from round to oval in shape. Numerous blood vessels were also seen. Immunohistochemical analysis of the granular cells revealed positivity for CD68, D2-40, Ki67, VEGF, and FGF and negativity for S100, CD34, and CD105. Immunostaining for CD34, CD105, and D2-40 confirmed the presence of a large number of blood and lymphatic vessels. Although rare, an understanding of this lesion is paramount for correct diagnosis and appropriate treatment. In the present report, the immunohistochemical profile confirmed increased vascularization, proving that these lesions are composed of not only new and preexisting blood vessels, but also lymphatic vessels.

## 1. Introduction

According to the most recent classification by the World Health Organization (WHO) [1], congenital granular cell epulis (CGCE), also known as congenital granular cell tumor, congenital epulis, congenital epulis of the newborn, congenital granular cell lesion, and gingival granular cell tumor of the newborn [2, 3], is a rare benign soft tissue lesion, which usually arises from the alveolar ridges of neonates. CGCE was first described in 1871 by Neumann, hence its original eponym being Neumann's tumor. CGCE is a sessile or pedunculated nodule, which is usually attached to the alveolar ridge of neonates, and although rare, the presence of multiple lesions is rarer still, having been reported in approximately 10% of cases [2].

Clinically, these lesions present as normal or red in color and vary in size, from several millimeters to a few centimeters. CGCE more commonly presents in females, near the midline of the anterior ridge, with the maxilla being more affected than the mandible at a ratio of 3 : 1. CGCE has not been associated with any other dental abnormality or congenital malformation [2–5]. Histologically, CGCE consists of large round granular cells with an abundant eosinophilic cytoplasm and basophilic nuclei set in a prominent vasculature. Cellular or nuclear pleomorphism is not observed [1]. Under the electron microscope, the cells of the epulis are seen to be packed with lysosomes, which further confirm the granular nature of the cells [6].

The present report describes the clinical, histopathological, and immunohistochemical profile of a case of multiple CGCE.

## 2. Case Report

**Figure 1:**
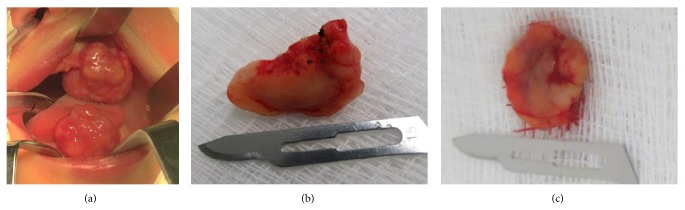
Clinical and macroscopic features. (a) Two pedunculated reddish nodules located in the mandible and maxilla. ((b) and (c)) The lesions following surgical excision.

A female Caucasian neonate presented two exophytic lesions of the mandible and maxilla, associated with feeding difficulties, described as pedunculated nodules, reddish in color, with an irregular surface, located at the anterior alveolar region between the future eruption sites of the incisors and canines, measuring approximately 3 cm (Figure 1(a)). Both lesions were similar, with nothing to distinguish one from the other. The clinical hypothesis was congenital granular cell epulis. The patient underwent surgical removal of the lesions under general anesthetic on the second day of life. The peri- and postoperative outcomes were satisfactory, with normal feeding established on the same day.

**Figure 2:**
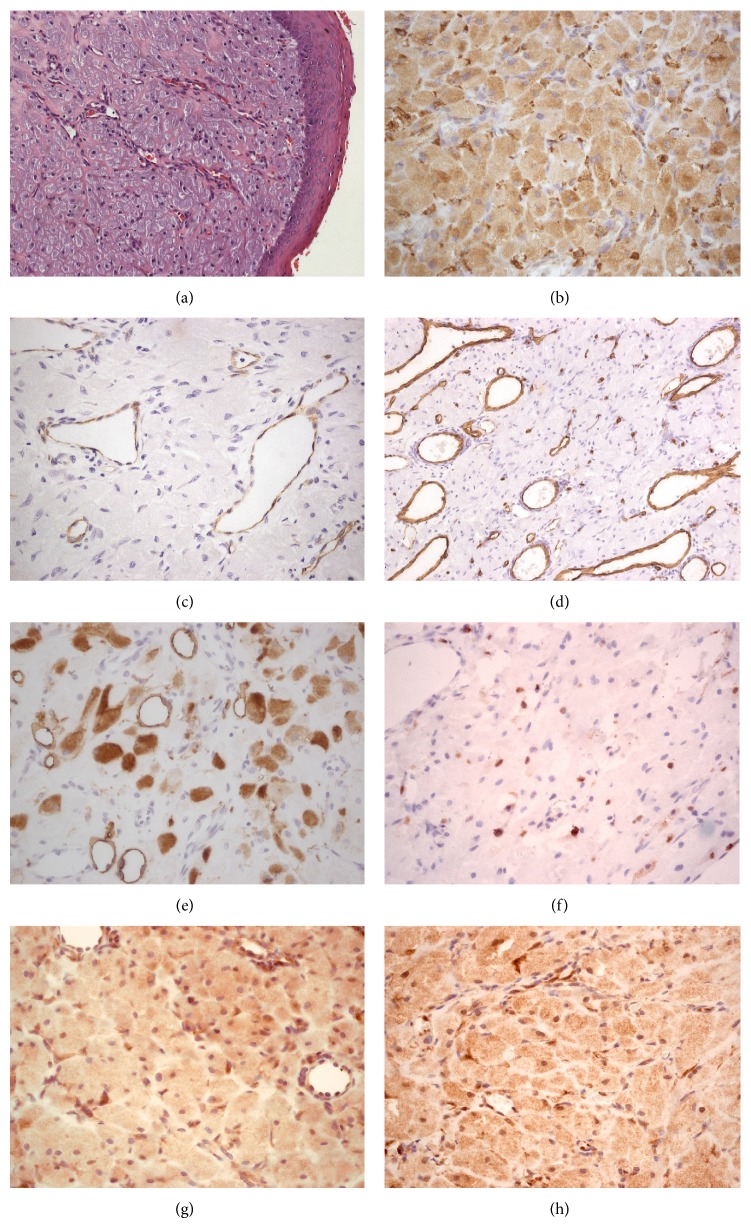
Histological and immunohistochemical sections. (a) Large round granular cells with an abundant eosinophilic cytoplasm and basophilic nuclei in a background of prominent vasculature and lined by an atrophic epithelium (H&E 200x). (b) Granular cells demonstrating strong positivity for CD68 (400x). ((c) and (d)) Endothelial cells positive for CD105 and CD34, respectively. (e) D2-40 positive immunostaining for granular cells and lymphatic vessels. (f) Weak staining for Ki67, showing a low grade of proliferation. ((g) and (h)) Overexpression of VEGF and FGF in granular cells and endothelial cells, respectively.

The specimens (Figures 1(b) and 1(c)) were fixed in 10% formalin and sent for histological examination. Microscopic examination of the hematoxylin and eosin (H&E) sections revealed both lesions to be similar, with fragments of oral mucosa lined by an atrophic parakeratinized stratified squamous epithelium observed. Proliferation of large round granular cells with an abundant eosinophilic cytoplasm and basophilic nuclei, ranging from round to oval, was observed in the lamina propria (Figure 2(a)). Increased vascularity composed of capillaries and small vessels was observed dispersed among the granular cells. The histopathologic diagnosis was congenital granular cell epulis. An immunohistochemical panel comprised of S-100, CD68, CD34, CD105, VEGF, FGF, D2-40, and Ki67 was performed (Table 1) in order to elucidate further histopathological characteristics. The granular cells were found to be negative for S-100, CD34, and CD105, yet positive for CD68, VEGF, FGF, D2-40, and Ki67 (Figures 2(b)–2(h)). Histopathological and immunohistochemical features were similar in both lesions. Wound healing following surgery was satisfactory, resulting in alveolar ridges of a normal appearance, with no recurrence reported.

## 3. Discussion

CGCE is a rare benign lesion, which occurs exclusively in the oral and maxillofacial region of neonates. Multiple lesions are infrequently described, with fewer than 30 case reports in the English literature. Generally, the lesions are located on one or both ridges or on a ridge and the tongue [2, 4–8].

This case report describes multiple CGCE of the maxilla and mandible, present at birth in a female newborn, which corroborates the clinical features described by other authors. Although the diagnosis of CGCE is usually clinical, due to its characteristic occurrence on the alveolar ridge in neonates, a differential diagnosis, which includes teratoma, hemangioma, lymphatic malformation, congenital malformation, or neoplasm, should also be considered [2, 5, 7].

The histological characteristics observed in this study were similar to those reported in the literature. Although other histological characteristics, such as the presence of spindle cells, fibrosis [3], and nests of odontogenic epithelium among the granular cells [1, 9], may also be present in CGCE, fibrosis and odontogenic epithelium were not observed in this study.

The principal histological differential diagnosis of CGCE is granular cell tumor (GCT). Both lesions are histologically similar; however, when considering the clinical, morphological, and immunohistochemical features together, it becomes possible to distinguish one from the other [3, 10, 11]. GCT is composed of large polygonal cells with an abundant granular eosinophilic cytoplasm arranged in layers, cords, or nests, with pseudoepitheliomatous hyperplasia of the epithelium and small peripheral nerves are often observed [12]. In addition, immunohistochemistry for GCT reveals positivity for S-100 [11]. In the present report, the diagnosis of GCT was excluded due to the clinical information, presence of an atrophic epithelium, and immunohistochemistry being negative for S-100.

Despite CGCE being first reported in the literature in 1871, its histogenesis remains controversial. A large number of theories have been proposed, consequently leading to immunohistochemical studies aimed at determining the origin of the granular cells [11–13]. In the current report, the granular cells were negative for S-100, CD34, and CD105 and positive for CD68, VEGF, FGF, D2-40, and Ki67.

The lack of immunoreactivity with S-100 suggests that CGCE is derived from a different cell line to GCT. It also highlights the absence of Schwann cells in CGCE, which corroborates the findings of other studies [2, 4–7, 12]. Other studies have, however, reported positivity for neuron-specific enolase (NSE), suggesting that a neural origin should not be ruled out [10].

Granular cells have been reported as positive for CD68 in not only GCT, but also some CGCE [11, 13]. In the present case, the polygonal cells were positive for CD68, which corroborates the findings of Kaiserling et al. [11], Lapid et al. [14], and Abo-Hager et al. [13], while contradicting authors who have reported negativity [3–5, 12]. Contradictory data may have arisen due to the rarity of the lesion, with most studies having been performed on a maximum of one or two lesions [12].

Increased vascularity is a common feature to CGCE, which can be confirmed both histologically and clinically, the latter being due to its reddish color. Some lesions have also had their blood flow observed during prenatal screening via color Doppler imaging [15, 16]. In the present study, immunohistochemical staining with CD34 (a panendothelial marker) and CD105 (a marker of neoangiogenesis) revealed the presence of a large number of mature and newly formed vessels, respectively.

Angiogenesis, the neoformation of vessels from preexisting ones, is essential for tumor growth. CD105 is a protein expressed predominantly by proliferating endothelial cells [17]. In the present study, although the tumor cells did not demonstrate granular positivity for this marker, vast and intense immunostaining was observed in the vascular endothelium, which may have a direct relationship with the growth of the lesion. The presence of this tumor on prenatal ultrasound imaging studies performed between weeks 27 and 30 of gestation may indicate that these lesions develop throughout the later stages of pregnancy, during the latter part of the second or throughout the third trimester [14, 15]. Therefore, the presence of newly formed vessels, observed via CD105, may be directly related to the growth of this lesion in the latter stages of pregnancy, near the time of delivery.

In this study, the granular cells were positive for podoplanin (clone D2-40), which has not previously been demonstrated for CGCE. Podoplanin expression has been reported in a variety of normal tissues, including lymphatic endothelial cells, mesothelial cells, osteocytes, chondrocytes, osteoblasts, stromal reticular cells, and dendritic cells of lymphoid tissues, the choroid plexus epithelium and ependymal cells of the central nervous system, myoepithelial cells of breast and salivary glands, myofibroblasts, and skeletal muscle cells, in addition to various tumors [18]. In the present case, positivity was observed for the lymphatic endothelium, revealing a significant lymphatic presence throughout the lesion.

The aforementioned findings indicate that the increased vascularity observed for CGCE was in fact due to the presence of both blood and lymphatic vessels, suggesting their importance in both the development and maintenance of the lesion. This is the first report describing the presence of lymphatic vessels in CGCE.

Proliferation and angiogenesis are key processes in the biology of tissue growth. In the present case, a low number of cells were Ki-67 positive, suggesting minimal cell proliferation, which was unexpected as CGCE is considered a recently formed lesion. This result is in agreement with Kato et al. [10], who demonstrated a labeling index of 16.7% for CGCE granular cells.

VEGF and FGF are two very important growth factors for angiogenesis and vasculogenesis [19, 20]. In the present study, intense staining for these biomarkers confirmed the intense and increased vascularization of preexistent and newly formed vessels, as demonstrated by CD34, CD105, and D2-40.

To date, a standard treatment protocol for this tumor has not been established. Its growth ceases postpartum, where spontaneous regression has been observed [21, 22]. Conservative surgical excision is usually the treatment of choice [3, 4, 7, 14], and recurrence has not been reported. Conservative treatment followed by close clinical follow-up has been described for lesions which do not interfere with feeding and breathing [20]. In the present case, conservative surgical excision was preferred, owing to the size of the lesions, which were preventing satisfactory breastfeeding and mouth closure, in addition to parental concern. The prognosis of CGCE is good, owing to its benign behavior and growth, and the absence of recurrence following excision, even when incompletely removed. Malignant transformation has not been reported [7]. In the current report, clinical follow-up at one month revealed alveolar ridges with a normal appearance, with no signs of recurrence.

## 4. Conclusion

Congenital granular cell epulis is a benign lesion that occurs almost exclusively in the mucosa of the alveolar ridges of neonates, with the occurrence of multiple lesions being rare. The immunohistochemical profile for the present case confirmed increased vascularization, demonstrating that the lesion is composed of new and preexisting blood and lymphatic vessels, suggesting a possible influence on lesion development during the latter stages of pregnancy. Although rare, it is important that the dental surgeon has an adequate understanding of the lesion in order to establish an accurate diagnosis and appropriate treatment.

## Figures and Tables

**Table 1:** Details of the antibodies used for immunohistochemistry.

Specificity	Clone	Dilution	Source	Buffer (AR)
CD68	M0814	1 : 500	Dako^*^	Citrate
S-100	Z0311	1 : 1200	Dako^*^	—
CD34	QBEnd 10	1 : 50	Dako^*^	Citrate
CD 105	SNG	1 : 10	Dako^*^	Pepsin
D2-40	D2-40	1 : 200	Dako^*^	Tris-EDTA
FGF II	Sc-79	1 : 100	Santa Cruz^**^	Citrate
VEGF	Sc-7269	1 : 100	Santa Cruz^**^	Citrate
Ki-67	MIB-1	1 : 400	Dako^*^	Citrate

∗ is: Dako Corporation, Glostrup, Denmark.

∗∗ is: Santa Cruz Biotechnogy, Inc., Santa Cruz, CA, USA.

## References

[1] Van der Waal I., Barnes L., Eveson J. W., Reichart P., Sidransky D. (2005). Congenital granular cell epulis. *World Health Organization Classification of Tumours. Pathology and Genetics. Head and Neck Tumours*.

[2] Dzieniecka M., Komorowska A., Grzelak-Krzymianowska A., Kulig A. (2011). Multiple congenital epuli (congenital granular cell tumours) in the newborn: a case report and review of literature. *Polish Journal of Pathology*.

[3] Conrad R., Perez M. C. N. (2014). Congenital granular cell epulis. *Archives of Pathology and Laboratory Medicine*.

[4] Lee J.-M., Kim U.-K., Shin S.-H. (2013). Multiple congenital epulis of the newborn: a case report and literature review. *Journal of Pediatric Surgery Case Reports*.

[5] Childers E. L. B., Fanburg-Smith J. C. (2011). Congenital epulis of the newborn: 10 new cases of a rare oral tumor. *Annals of Diagnostic Pathology*.

[6] Mirchandani R., Sciubba J. J., Mir R. (1989). Granular cell lesions of the jaws and oral cavity: a clinicopathologic, immunohistochemical, and ultrastructural study. *Journal of Oral and Maxillofacial Surgery*.

[7] Damante J. H., de Souza Tolentino E., Mazzottini R., Monteiro-Amado F., Fleury R. N., Soares C. T. (2011). Congenital granular cell lesion: clinical, microscopic and immunohistochemical aspects in a case of multiple lesions. *Journal of Clinical Pediatric Dentistry*.

[8] Loyola A. M., Gatti A. F., Santos Pinto D., Mesquita R. A. (1997). Alveolar and extra-alveolar granular cell lesions of the newborn: report of case and review of literature. *Oral Surgery, Oral Medicine, Oral Pathology, Oral Radiology, and Endodontics*.

[9] Godra A., D'Cruz C. A., Labat M. F., Isaacson G. (2004). Pathologic quiz case: a newborn with a midline buccal mucosa mass. *Archives of Pathology and Laboratory Medicine*.

[10] Kato H., Nomura J., Matsumura Y., Yanase S., Nakanishi K., Tagawa T. (2013). A case of congenital granular cell epulis in the maxillary anterior ridge: a study of cell proliferation using immunohistological staining. *Journal of Maxillofacial and Oral Surgery*.

[11] Kaiserling E., Ruck P., Xiao J.-C. (1995). Congenital epulis and granular cell tumor. A histologic and immunohistochemical study. *Oral Surgery, Oral Medicine, Oral Pathology, Oral Radiology and*.

[12] Vered M., Dobriyan A., Buchner A. (2009). Congenital granular cell epulis presents an immunohistochemical profile that distinguishes it from the granular cell tumor of the adult. *Virchows Archiv*.

[13] Abo-Hager E. A., Khater D. S., Ahmed M. M. (2009). Exploration of the histogenesis of congenital granular cell epulis: an immunohistochemical study. *Journal of the Egyptian National Cancer Institute*.

[14] Lapid O., Shaco-Levy R., Krieger Y., Kachko L., Sagi A. (2001). Congenital epulis. *Pediatrics*.

[15] Jiang L., Hu B., Guo Q. (2011). Prenatal sonographic diagnosis of congenital epulis. *Journal of Clinical Ultrasound*.

[16] Kim S.-K., Won H.-S., Lee S. W. (2006). Prenatal diagnosis of congenital epulis by three-dimensional ultrasound and magnetic resonance imaging. *Prenatal Diagnosis*.

[17] Duff S. E., Li C., Garland J. M., Kumar S. (2003). CD105 is important for angiogenesis: evidence and potential applications. *FASEB Journal*.

[18] Ordóñez N. G. (2014). Value of podoplanin as an immunohistochemical marker in tumor diagnosis: a review and update. *Applied Immunohistochemistry and Molecular Morphology*.

[19] Olsson A.-K., Dimberg A., Kreuger J., Claesson-Welsh L. (2006). VEGF receptor signalling—in control of vascular function. *Nature Reviews Molecular Cell Biology*.

[20] Turner N., Grose R. (2010). Fibroblast growth factor signalling: from development to cancer. *Nature Reviews Cancer*.

[21] Bhatia S. K., Goyal A., Ritwik P., Rai S. (2013). Spontaneous regression of a congenital epulis in a newborn. *Journal of Clinical Pediatric Dentistry*.

[22] Ritwik P., Brannon R. B., Musselman R. J. (2010). Spontaneous regression of congenital epulis: a case report and review of the literature. *Journal of Medical Case Reports*.

